# Measuring quality of life in people living with and beyond cancer in the UK

**DOI:** 10.1007/s00520-021-06105-z

**Published:** 2021-03-30

**Authors:** Elisavet Moschopoulou, Jennifer Deane, Morvwen Duncan, Sharif A. Ismail, Sophie Moriarty, Shah-Jalal Sarker, Peter White, Ania Korszun, on behalf of the SURECAN Development Grant investigators, Kamaldeep Bhui, Liam Bourke, Trudie Chalder, Sandra Eldridge, John Gribben, Louise Jones, Paul McCrone, Adrienne Morgan, Damien Ridge, Rebecca Roylance, Steph Taylor, Mohamed Thaha

**Affiliations:** 1 Institute of Population Health Sciences, Barts and The London School of Medicine and Dentistry, Queen Mary University of London, London, UK; 2 Population Health Sciences Institute, Newcastle University, Baddiley-Clark Building, Newcastle upon Tyne, NE2 4AX UK; 3 Centre for Psychiatry, Wolfson Institute of Preventive Medicine, Queen Mary University of London, Charterhouse Square, London, EC1M 6BQ UK; 4 Department of Global Health and Development, London School of Hygiene and Tropical Medicine, 15-17 Tavistock Place, London, WC1H 9SH UK; 5 Research Department of Medical Education, UCL Medical School, UCL, London, UK

**Keywords:** Quality of life, Cancer, Survivorship, Assessment

## Abstract

**Purpose:**

The aim of this study was to identify the most appropriate measure of quality of life (QoL) for patients living with and beyond cancer.

**Methods:**

One hundred eighty-two people attending cancer clinics in Central London at various stages post-treatment, completed a series of QoL measures: FACT-G, EORTC QLQ-C30 , IOCv2 (positive and negative subscales) and WEMWBS, a wellbeing measure. These measures were chosen as the commonest measures used in previous research. Correlation tests were used to assess the association between scales. Participants were also asked about pertinence and ease of completion.

**Results:**

There was a significant positive correlation between the four domain scores of the two health-related QoL measures (.32 ≤ *r* ≤ .72, *P* < .001), and a significant large negative correlation between these and the negative IOCv2 subscale scores (− .39 ≤ *r* ≤ − .63, *P* < .001). There was a significant moderate positive correlation between positive IOCv2 subscale and WEMWBS scores (*r* = .35, *P* < .001). However, neither the FACT-G nor the EORTC showed any significant correlation with the positive IOCv2 subscale. Participants rated all measures similarly with regards to pertinence and ease of use.

**Conclusion:**

There was little to choose between FACT-G, EORTC, and the negative IOC scales, any of which may be used to measure QoL. However, the two IOCv2 subscales capture unique aspects of QoL compared to the other measures. The IOCv2 can be used to identify those cancer survivors who would benefit from interventions to improve their QoL and to target specific needs thereby providing more holistic and personalised care beyond cancer treatment.

## Introduction

By 2015, there were an estimated 2.5 million people in the UK previously diagnosed with cancer [1], and rates continue to rise with prediction of increasing survival rates for most cancers [2, 3]. It is projected that there will be 4 million people living with or beyond cancer by 2030 [4]. As treatments and survival continue to improve, cancer now represents a chronic life-altering condition, and quality of life (QoL) is increasingly recognised as an important treatment outcome for those living with cancer [5, 6]. Depending on the type of cancer, survivors can experience a variety of adverse and late physical effects of cancer therapy, such as fatigue, pain and disability. However, survivors themselves have identified social and psychological problems (including depression, anxiety, and fear of recurrence) as important determinants of their QoL [7]. Studies have also shown that such psychosocial factors are more highly associated with QoL than the type and extent of the cancer. Although these problems are clearly prioritised by cancer patients [8], they still receive comparatively less attention in clinical care and research. A 2019 British study of 526 colorectal cancer survivors found that unmet psychological needs were the highest of all unmet needs following treatment [9]. These psychological unmet needs included feelings of sadness, loss of control, fear of cancer recurrence and death, uncertainty, and difficulty in keeping a positive outlook. Notably, the percentage of patients with psychological unmet needs, 15 months after treatment, remained consistent when measured again at 24 months. These unmet needs, in addition to physical, health system, and informational needs, were associated with poorer health-related quality of life at the end of treatment. Therefore, it is potentially possible to identify ‘at risk’ patients as soon as they have finished treatment [9].

Defining and measuring the QoL of people living with and beyond cancer is not straightforward. There are several well validated and widely used measures of health-related QoL, but these focus predominantly on physical symptoms (rather than psychosocial issues) related to the functional effects of being treated for cancer, and often include specific symptoms such as shortness of breath or disabilities such as problems walking. Such symptoms can also be related to other comorbid conditions and, in a recent study, Vissers and colleagues [10] showed that medical comorbidity explained more variance in health-related QoL than did cancer characteristics. Furthermore, the study of cancer survivorship abounds with studies examining primarily negative outcomes such as distress, diminished QoL and functional impairments; less is known about positive changes following the experience of cancer. A more contemporary approach to the study of QoL acknowledges the multidimensional and dynamic impact of cancer whereby the experience of cancer can be life changing in both negative and positive ways [11].

The Impact of Cancer (IOCv2) questionnaire was specifically developed to measure not only the unique negative impacts of cancer, such as worry and body change concerns, but also the positive impact of cancer associated with long-term survivorship, such as increased altruism and positive self-evaluation [12]. This measure has been used in studies of breast and lymphoma cancer survivors in the USA and Europe [13–16]. In our 2014 study of British haematological cancer survivors [7], we found that levels of depression and psychological distress in this survivor group were three times higher than in the general population, and these symptoms were significantly associated with more negative IOCv2 scores. On the other hand, the type and stage of the cancer, or whether there had been a recurrence of the cancer, had no relationship to IOCv2 scores. Positive IOCv2 scores showed a distinctive pattern of association that reflected factors such as level of education and social support. The IOC was originally developed for long-term survivors (> 2 years after diagnosis); however, many of the issues addressed in this measure are also experienced by those at earlier stages in the cancer pathway.

Two of the most commonly used cancer-specific health-related QoL measures are the European Organisation for Research and Treatment for Cancer Quality of Life Questionnaire Core 30 (EORTC QLQ-C30) [17], and the Functional Assessment of Cancer Therapy—General Scale (FACT-G) [18]. These instruments are usually combined with questionnaires assessing the impact of the specific type of cancer. Although both measures have high validity and reliability, they have been shown to have different applications [19]. For instance, the EORTC focuses more on disease symptoms and consequences of treatment, whereas the FACT-G is more advantageous at assessing emotional aspects. Nevertheless, these questionnaires may not address some of the more subtle aspects of QoL identified by those living with and beyond cancer as they were designed to focus primarily on the early stages of being diagnosed with and treated for cancer [20]. As patients move towards the survivorship phase, although certain functional and physical impairments remain, other issues may become equally or more relevant. Therefore, these instruments may not adequately capture the unique needs of longer-term cancer survivors.

The IOC was originally developed using qualitative methodology to explore the constructs of both negative and positive impacts of cancer. Interviews were carried out with a sample of patients who had survived cancer for 5 or more years, but were entirely based in Los Angeles, USA [12]. While a proportion of participants were non-White, there was no information about the ethnicities involved. It was unclear how useful instruments like the IOCv2 were when used by British patients, and with more ethnically mixed groups of patients in the UK, until a 2017 study compared the use of IOCv2 in non-Hodgkin lymphoma (NHL) survivors in USA and UK populations. Good reliability of the IOCv2 was found, and so it was deemed potentially applicable to UK populations [21]. In addition, this study demonstrated that psychosocial factors had a greater impact on QoL than disease characteristics [21]. With the increasing focus on how to improve QoL for patients, an appropriate outcome measure is a priority for research. Better conceptualization of QoL for people living with and beyond cancer, and identification of useful measures that reflect UK patients’ realities, needs, and perceptions have become pressing.

The specific objectives of this study were to compare commonly used QoL measures (FACT-G and EORTC QLQ-C30) across corresponding domains (physical, emotional, social and functional); as well as to the IOCv2 scale (positive and negative subscales) and to an overall wellbeing measure (WEMWBS) in different populations of cancer patients who had completed active treatment. We did not seek correlations against the EORTC QLQ-C30 cognitive functioning sub-scale, since there was no equivalent domain in the FACT-G questionnaire. The aim was to determine the optimal method to identify and measure QoL in cancer patients living with and beyond cancer. The hypotheses under test were:

i. Corresponding domains in FACT-G and EORTC QLQ-C30 are positively correlated and there is a negative correlation with negative (–ve) IOCv2 as well as a positive correlation with WEMWBS.
ii. FACT-G and EORTC QLQ-C30 do not correlate with positive (+ve) IOCv2.

## Methods

This study was carried out as a clinical audit (“Audit of well-being/quality of life measures in cancer survivors” ID: 5711(2015) Barts Health NHS Trust) in oncology outpatient clinics throughout Barts Health NHS Trust. A clinical audit aims to evaluate the quality of current practice regarding a specific aspect of healthcare and to identify any changes needed in order to improve the care provided to patients. Therefore, research projects and clinical audit projects serve different purposes with the latter forming part of the continuous quality improvement process [22].

### Inclusion and exclusion criteria

Patients were eligible if they had finished active treatment for cancer and were over the age of 18. Patients were excluded if they were not able to speak English or if they were receiving palliative care. Patients attending cancer clinics in central London were screened against the eligibility criteria by a member of the clinical care team. Eligible patients were then approached at the clinic by a research assistant who invited them to complete a series of questionnaires and provided them with the study information sheet. No financial incentives or any other forms of compensation were used. The clinics attended were breast, head and neck, colorectal and haematology-oncology (late effects and chronic myeloid leukaemia (CML), myeloma, and the leukaemia and lymphoma clinics).

### Measures

*FACT-G* is a 27-item generic HRQL measure that has been widely validated [18]. Each item has 5 options ranging from *not at all* (a score of 0) to *very much* (a score of 4), and these are summed to obtain a global score, as well as 4 subscale scores: physical well-being, social/family well-being, emotional well-being, and functional well-being. A higher score signifies better QoL.

*EORTC QLQ-C30* is a 30-item cancer-specific HRQL measure that has also been widely validated [23]. Two items ask about overall QoL and overall health, and the remainder covers 5 functioning scales (physical, role, social, emotional, and cognitive functioning) and 9 symptoms scales (fatigue, nausea and vomiting, pain, dyspnea, sleep disturbance, appetite loss, constipation, diarrhoea, and financial impact). A higher score for the global QoL or the functioning scales signifies better QoL.

*Warwick–Edinburgh Wellbeing Scale (WEMWBS)* [24] measures a broad concept of mental wellbeing, including positive affect, psychological functioning (autonomy, competence, self-acceptance, personal growth), and interpersonal relationships. A higher score signifies better QoL. The WEMWBS was chosen as it focuses on positive aspects of mental health, it is valid in a range of settings and it can be used at a population level or with targeted clinical groups. Furthermore, it is relatively short and easy to complete compared to the other measures included in this study.

*Impact of Cancer V2 (IOCv2)* is a 37-item shortened version of the IOC [25]. The IOCv2 measures the unique positive and negative impacts of cancer associated with long-term survivorship. Participants rate their level of agreement for a given statement from 1 (strongly disagree) to 5 (strongly agree), which results in both a positive and negative scale. The scales are subdivided into eight subscales. The four positive subscales include: Altruism/Empathy, Health Awareness, Meaning of Cancer and Positive Self-Evaluation; a higher score signifies better QoL. The negative subscales include Appearance Concerns, Body Change Concerns, Life Interferences and Worry. A higher score signifies worse QoL.

In addition, patients were asked to rate each questionnaire on a 5-point Likert scale with regards to (a) how much it addressed their issues and (b) how easy it was to complete. Sociodemographic data were also collected.

### Statistical analysis

Data were analysed using statistical software package SPSS version 24. Missing data were not imputed. Spearman’s rank correlation was used as the data were not normally distributed. One-tailed tests were used to predict correlation across the questionnaires according with our one-directional hypotheses. A *P* value of less than 0.01 was considered statistically significant. We chose this conservative *P* value due to the presence of multiplicity. The medians (interquartile range) of Likert score responses to the two questions about pertinence and ease of completion were calculated. Friedman’s test was used to detect any statistically significant differences in the pertinence and ease of completion ratings between the four different measures. Cronbach’s alpha was calculated for each measure in the study in order to assess internal consistency. Further analysis was conducted to compare QoL scores for each measure according to cancer diagnosis using Kruskal-Wallis test with Dunn’s post hoc test. Alpha coefficient values suggested that all measures were fit for purpose (IOC positive α = 0.85; IOC negative α = 0.89; WEMWBS α = 0.93; FACT-G α = 0.92; EORTC QLQ-C30 α = 0.88).

## Results

**Fig. 1:**
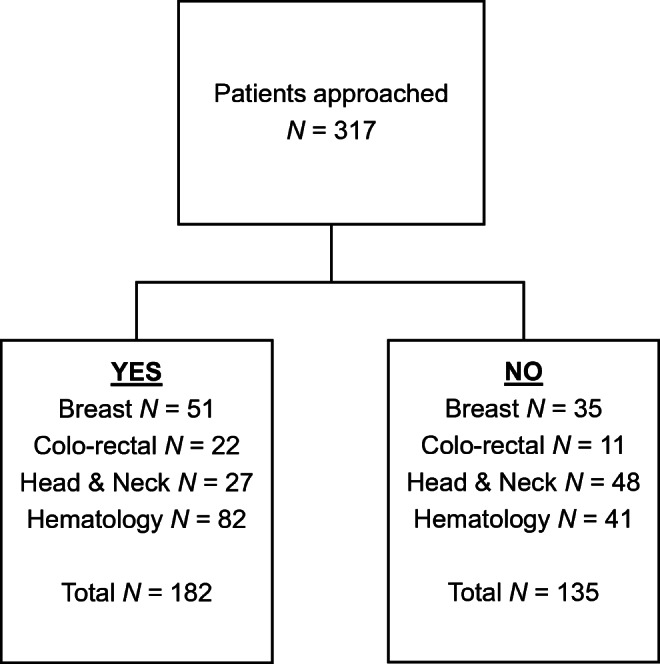
Cancer types of completers vs non-completers

Of the 317 patients who were approached, 182 agreed to participate in the audit. Therefore, the response rate was 57%. Reasons were recorded for those who declined to take part. Language barriers and lack of time were the most commonly cited issues. In this audit, information was available only on gender and cancer type. Figure 1 shows the completers vs non-completers by cancer type.

There was no significant difference in gender between completers and non-completers. However, those with head and neck cancer were more likely to decline to take part.

The demographics of participants are shown in Table 1: 57% of participants were women and 77% of participants were white. Age ranged from 18 to > 75 years.

**Table 1:** Socio-demographic characteristics of participants in the audit (*N* = 182)

Characteristics	Frequency	Percent
*Ethnicity (n = 178, missing = 4)*		
White	141	79.2
Asian	16	9
Black	16	9
Mixed	3	1.7
Chinese	2	1.1
*Age category (n = 175, missing = 7)*		
18–24	3	1.7
25–34	5	2.9
35–44	23	13.1
45–54	34	19.4
55–64	55	31.4
65–74	35	20
75+	20	11.4
*Gender (N = 180, missing = 2)*		
Male	77	42.8
Female	103	57.2
*Clinic attended (n = 182)*		
Colorectal	22	-
Breast	51	-
Haematology–leukaemia	25	-
Haematology–late effects	27	-
Haematology–lymphoma	9	-
Haematology–multiple myeloma	21	-
Head and neck	27	-
*Education level (n = 156, missing = 26)*		
Degree	51	32.7
Technical qualification	22	14.1
A levels or equivalent	18	11.5
GCSE’s, O’ levels or equivalent	46	29.5
Other	19	12.2
*Relationship status (n = 175, missing = 7)*		
Partnered	103	58.9
Non-partnered	72	41.1

Table 2 shows the correlations between scores on the different measures. As hypothesised, QLQ-C30 and FACT-G significantly positively correlated with each other across corresponding domains: physical, social, emotional, and functional respectively (.32 ≤ *r* ≤ .72, *P* < .001). The strongest correlation was between the physical sub-scales of the two questionnaires (*r* = .74, *P* < .001), whereas the association between the social sub-scales was the weakest (*r* = .32, *P* < .001). Furthermore, each domain of both measures was significantly negatively correlated with negative IOC (− .39 ≤ *r* ≤ − .63, *P* < .001), i.e. high negative impact was correlated with poorer QoL. The social and emotional domains of the EORTC QLQ-C30 were most strongly correlated with negative IOC (*r* = − .57, *P* < .001 for both), whereas regarding the FACT-G, the strongest correlation with negative IOC was for the physical domain (*r* = − .63, *P* < .001). The IOC positive measure generally did not correlate across any of the QoL measures. There was a significant moderately positive correlation between WEMWBS and positive IOC scores (*r* = .35, *P* < .001). Regarding correlations between total scores, the strongest correlations were between the FACT-G and the Global QoL of the EORT QLQ-C30 (*r* = .76, *P* < .001), as well as between FACT-G and WEMWBS total scores (*r* = .72, *P* < .001). There were no statistically significant correlations between the IOC positive scale score and other total scores except for the aforementioned positive correlation with WEMWBS. The IOC negative total score correlated highly with both the FACT-G total score (*r* = − .70, *P* < .001) and with the Global QoL score of the EORTC QLQ-C30 (*r* = − .54, *P* < .001).

**Table 2:** Associations of the sub-domains and the total scores of the questionnaires

		IOC positive impact scale score	IOC negative impact scale score	WEMWBS total score	EORTC QLQ-C30	FACT-G
		*r*, *n*, *P* value	*r*, *n*, *P* value	*r*, *n*, *P* value	*r*, *n*, *P* value	*r*, *n*, *P* value
2a: Association of the physical domains of EORTC and FACT-G	IOCV2 positive impact scale score	1, 151, –				
	IOCV2 negative impact scale score	.04,140, .333	1, 150, –			
	WEMWBS total score	.35, 146, < .001	− .52, 147, < .001	1, 174, –		
	EORTC QLQ-C30	− .007, 148, .468	− .44, 148, < .001	.39, 170, < .001	1,177,-	
	FACT-G	.010, 150, .453	− .63, 150, < .001	.50, 173, < .001	.74, 176, < .001	1, 179, –
2b: Association of the social domains of EORTC and FACT-G	IOC positive impact scale score	1, 151, –				
	IOC negative impact scale score	.04,140, .333	1, 150, –			
	WEMWBS total score	.35, 146, < .001	–.52, 147, < .001	1, 174, –		
	EORTC QLQ-C30	.01, 146, .442	− .57, 146, < .001	.44, 167, < .001	1, 174, -	
	FACT-G	.21, 143, .006	− .51, 145, < .001	.56, 166, < .001	.32, 165, < .001	1, 171, –
2c: Association of the emotional domains of EORTC and FACT-G	IOC positive impact scale score	1, 151, –				
	IOC negative impact scale score	.04,140, .333	1, 150, –			
	WEMWBS total score	.35, 146, < .001	− .52, 147, < .001	1, 174, –		
	EORTC QLQ-C30	.29, 147, .366	-.57, 147, <.001	.52, 169, <.001	1, 176, -	
	FACT-G	− .02, 149, .397	− .56, 150, < .001	.39, 168, < .001	.55, 170, < .001	1, 174, –
2d: Association of the functional domains of EORTC and FACT-G	IOC positive impact scale score	1, 151, –				
	IOC negative impact scale score	.04,140, .333	1, 150, –			
	WEMWBS total score	.35, 146, < .001	− .52, 147, < .001	1, 174, -		
	EORTC QLQ-C30	.01, 148, .436	− .39, 147, < .001	.39, 170, < .001	1, 176, –	
	FACT-G	.19, 147, .011	− .55, 149, < .001	.67, 166, < .001	.56, 168, < .001	1, 172, –
2e: Association of total scores ^a^	IOCV2 positive impact scale total score	1, 151, –				
	IOCV2 negative impact scale total score	.04,140, .666	1, 150, –			
	WEMWBS total score	.35, 146, < .001	− .52, 147, < .001	1, 174, -		
	EORTC QLQ-C30 Global QoL score	.09, 143, .315	− .54, 142, < .001	.59, 159, < .001	1,164, –	
	FACT-G total score	.13, 143, .123	− .70, 147, < .001	.72, 162, < .001	.76, 155, < .001	1, 167, –

*Note. r* Spearman’s rank correlation coefficient, *n* sample size, *IOC* impact of cancer, *WEMWBS* Warwick-Edinburgh Mental Wellbeing Scale, *EORTC QLQ-C30* European Organisation for Research and Treatment of Cancer–Quality of Life Questionnaire, *FACT-G* Functional Assessment of Cancer Therapy–General

^a^Correlations between total scores are significant at the 0.05 level (two-tailed)

There was no evidence of a significant difference in QoL scores among the cancer groups at the considered 1% level of significance. However, at the conventional 5% level of significance, EORTC Global QoL scale score may be considered differed among the cancer groups (Table 3). A Kruskal-Wallis test showed there was a significant difference (*P* = .043) between at least one pair of groups. Dunn’s pairwise tests confirmed (adjusted using the Bonferroni correction) that the significant difference (*P* = .028) was between breast and colorectal patients.

**Table 3:** Total QoL scores for five scales according to cancer diagnosis

	IOC positive	IOC negative	WEMWBS	EORTC	FACT-G
	Median	Median	Median	Median	Median
Breast	59	57	51	66.7	12.6
Colorectal	59	50	55	83.3	13.4
Head and neck	62	50	53	75	13.5
Haematology	61	61	54	75	12.4
Kruskal-Wallis test (*P* value)	0.98 (0.81)	7.29 (0.06)	5.12 (0.16)	8.15 (0.04)	3.11 (0.37)

*Note: IOC* impact of cancer, *WEMWBS* Warwick-Edinburgh Mental Wellbeing Scale, *EORTC QLQ-C30* European Organization for Research and Treatment of Cancer–Quality of Life Questionnaire, *FACT-G* Functional Assessment of Cancer Therapy-General

The median scores and the interquartile range of the two additional questions regarding pertinence and ease of completion can be seen in Table 4. Scores were similar for all the questionnaires with the FACT-G scoring the highest on the ease of completion question. Nevertheless, a Friedman test indicated the four scales were not rated significantly differently on either question (pertinence: *χ*^2^ (3) = 0.63, *P* = 0.89; ease of completion: *χ*^2^ (3) = 3.99, *P* = 0.26).

**Table 4:** Medians and Inter-quartile range of Q1 and Q2 Final Questionnaire

	How well did it address your issues? (Q1)	How easy is it to complete? (Q2)
	Median (inter-quartile range)	Median (inter-quartile range)
FACT-G	4 (1)	5 (1)
QLQ-C30	4 (1)	4 (1)
WEMWBS	4 (1)	4 (1)
IOCV2	4 (1)	4 (1)

*Note: IOC* impact of cancer, *WEMWBS* Warwick-Edinburgh Mental Wellbeing Scale, *EORTC QLQ-C30* European Organization for Research and Treatment of Cancer–Quality of Life Questionnaire, *FACT-G* Functional Assessment of Cancer Therapy-General

## Discussion

In this study of patients attending a range of cancer clinics in London, we compared two widely used QoL measures (FACT-G and EORTC QLQ-C30) and a general wellbeing scale (WEMWBS) with the IOC scale, designed specifically for cancer survivors to address both negative and positive aspects of living with and beyond cancer.

There were positive correlations between the two health-related QoL subscale measures of the same domains (physical, social, emotional, and functional of FACT-G and EORTC QLQ-C30), as well as between the total FACT-G score and the Global QoL subscale score of the EORTC QLQ-C30. As hypothesised, a negative correlation between the two health-related QoL measures and the negative IOC scale was also observed. There was also a positive correlation between positive IOC and the WEMWBS general wellbeing measure; though WEMWBS is not specifically applicable to participants’ cancer experience. Neither the FACT-G nor the EORTC QLQ-C30 showed any correlation with the positive IOC scale, suggesting that the positive IOC scale measures something different from health-related QoL. Finally, of the QoL measures used in this audit, the participants reported similar ease of completion and preference for all the scales.

The analyses show that the IOC positive scores did not correlate with, and, therefore, were not covered by any of the other QoL measures, except for a moderate correlation with the WEMWBS scale, which was to be expected as that scale too focuses on positive aspects of general well-being. The IOC is therefore not only highly relevant to cancer patients in a central London clinic, but also unique amongst other scales in addressing the more positive aspects of perceived QoL in the post-treatment phase.

There is evidence that positive IOC is not merely the obverse of negative IOC, but represents different constructs of cancer survivors’ experience that translate across different countries and contexts [21]. Positive IOC may reflect a less understood area of cancer enquiry: that some patients, for reasons which are not entirely clear, are able to educe positive experiences from a significant and stressful life event such as being diagnosed with and treated for cancer.

Importantly, this ability may be a skill that could be acquired and there are promising therapies available such as Acceptance Commitment Therapy (ACT), which focus on helping patients to re-engage with what matters to them the most, thus promoting a values-based living while at the same time working on acceptance of things that are not within one’s control. Although a higher positive IOC score may not necessarily translate into a better functional level, people living with cancer might experience a better QoL, which is an important outcome. A 2015 study showed that both positive and negative perceptions of the impact of cancer are independent mediators of QoL [13]. Future studies could examine the effectiveness of using ACT and other supportive interventions to promote positive cancer outcomes for patients in the survivorship phase, as well as for patients under active treatment whenever possible.

### Study limitations

A limitation of this study was the 57% participation in the audit, which is consistent with response rates in similar studies [21]. The reasons for non-participation of patients are instructive for future research, e.g. some patients felt overwhelmed by the need to fill in questionnaires, some had problems with literacy, and many did not have enough command of the English language to participate. There was also a preponderance of White British participants and thus these results may not generalise to other groups. However, this also highlights the need for a simple, quick, and easily understood questionnaire that can be used as an effective screening method.

## Conclusion

Determining how best to measure QoL in people living with and beyond cancer is of paramount importance to ensure the best approach to screening and assessing the effectiveness of interventions but also to ensure holistic care within clinics that extends beyond cancer treatment. Our results suggest that both the FACT-G and the EORTC QLQ-C30 are measuring similar aspects of QoL and are equally pertinent and easy to use; so either may be used. The current findings suggest that the IOC captures a unique aspect of QoL over the other measures, related to post-traumatic growth [26].

## Data Availability

The datasets generated during and/or analysed during the current study are available from the corresponding author on reasonable request.
